# Prediction of Modulus of Elasticity of Concrete Using Different Homogenization Methods

**DOI:** 10.3390/ma18122674

**Published:** 2025-06-06

**Authors:** Jing Zhou, Hang Lin, Kaishun Qiu, Ke Ou, Fenghua Nie

**Affiliations:** 1 School of Resources and Safety Engineering, Central South University, Changsha 410083, China; 2 Department of Civil and Environmental Engineering, Imperial College London, London SW7 2BU, UK; 3 China Railway No. 5 Engineering Group Co., Ltd., Changsha 410100, China

**Keywords:** concrete, elastic modulus, mesomechanics, Mathematica, homogenization

## Abstract

Concrete is a highly heterogeneous composite material, and accurately predicting its elastic modulus remains a major challenge in mechanical analysis. To address this, this study systematically investigates the predictive performance of several classical homogenization methods for estimating the effective elastic modulus of concrete, including the dilute approximation, self-consistent method, generalized self-consistent method, Mori–Tanaka model, differential method, as well as the Voigt and Reuss models. To enhance prediction accuracy, an improved computational framework is proposed based on an iterative strategy that enables dynamic updating of model parameters. This approach combines principles of mesomechanics with numerical simulation techniques and is implemented using Mathematica for both symbolic and numerical computations. The performance of the models is evaluated under varying aggregate volume fractions and aggregate-matrix stiffness combinations, and validated using multiple experimental datasets from the literature. The results show that the iterative strategy significantly improves the predictive accuracy of several models, reducing the maximum error by up to 30%. Further analysis indicates that the dilute method performs best at low aggregate volume fractions, the Mori–Tanaka model yields the most accurate results when the aggregates are stiff and moderately concentrated, and the generalized self-consistent method outperforms the standard version when the elastic moduli of the aggregate and matrix are similar.

## 1. Introduction

Concrete is currently the most consumed man-made construction material in the world, and its applications are continuing to expand. In 2020, 14 billion cubic meters of concrete were consumed globally, and demand is still growing [1,2]. Concrete is a composite material composed of many different materials, including coarse aggregates, fine aggregates, colloids formed after cement hydration, cement particles that are not involved in hydration, as well as holes and cracks within the material. The complexity of these components and their interactions result in concrete exhibiting a high degree of variability in its physical and mechanical properties. For example, the bulk density of normal concrete typically ranges from 2300 kg/m^3^ to 2500 kg/m^3^, with corresponding elastic moduli ranging from 20,000 MPa to 35,000 MPa. In contrast, lightweight concrete, such as foam concrete, has a much wider range of bulk densities, from 300 kg/m^3^ to 1600 kg/m^3^, and corresponding elastic moduli ranging from 1000 MPa to 2000 MPa [3]. Enhancing one property of concrete may lead to a decrease in other properties, reflecting the intrinsic law of mutual constraints between material properties [4,5].

The elastic modulus is an important property of concrete materials, which reflects the stiffness and elastic properties of the material [6]. The elastic modulus of concrete should be obtained first in the calculation of deformation and cracking of concrete structures [7,8]. Therefore, accurate assessment of the elastic modulus of concrete plays a key role in structural design, optimization of material properties, and durability assessment. The internal structure of concrete can be viewed at three levels: macro-level, meso-level, and micro-level [9,10,11]. Some early studies treated concrete as an isotropic homogeneous material and used methods such as experiments or numerical simulations to study its elastic modulus and other macroscopic mechanical properties [12,13,14]. However, as far as the fine level is concerned, the distribution of individual components of concrete materials and their mechanical properties has a certain degree of randomness, and the fine structure influences the macroscopic mechanical properties and damage mechanisms. Neglecting the complex microstructure of concrete will make it difficult to reveal the deformation and damage mechanism of the material, and then it is difficult to construct an accurate material behavior model. A large number of experiments have shown that the damage phenomenon of concrete at the macroscopic level is actually caused by the gradual accumulation and development of damage and fracture at the microscopic level [15,16]. Thus, since the late 1970s [17], many scholars have begun to explore the nature of concrete damage from the fine level, In recent years, with the rapid advancement of computer technology and computational power, numerical simulation has been widely adopted in civil engineering as an efficient and controllable complement to traditional experimental methods [18,19,20]. Its integration with emerging technologies such as machine learning [21,22] has further expanded its application scope. In this context, increasing attention has been paid to combining meso-mechanical theories with numerical computation to enable in-depth analysis of the mechanical behavior of concrete at the meso scale. Various models of micromechanics consider concrete as a three-phase inhomogeneous composite formed by coarse aggregate, hardened cement colloid, and interfacial bonding between the two. As micromechanical models continue to be researched and developed, the most widely used models at present mainly include: lattice model [23,24], random aggregate model [25,26], random mechanical properties model [27], random particle model [28,29], micro planar model [30,31], etc. By selecting a suitable concrete fine mechanical structure model and applying numerical calculation techniques, the crack extension and damage process of concrete samples can be simulated.

Homogenization theory is a mathematically rigorous set of theoretical frameworks that have a place in contemporary research in applied mathematics. Over the past decade or so, there has been an increasing tendency to use homogenization methods to calculate equivalent material properties of composites at the macroscopic level, and this approach has gained popularity because of its effectiveness. For example, Peng, Y.J. et al. [32] applied the homogeneous mass method to investigate the strength and damage mechanism of recycled concrete materials were investigated using the homogeneous mass method to obtain the tensile strength, stress-strain softening curve and crack extension process of recycled concrete materials. Sivtsev et al. [33] simplified the complex nonlinear plastic deformation calculation process of fiber-reinforced concrete by applying the homogeneous mass method, which both ensures result accuracy and reduces computational complexity. Li, Y. et al. [34] classified concrete materials into four scales, from nanoscale to macroscale, to quantitatively assess creep properties. The creep modulus of concrete was then calculated using a homogenization method that combines the Mori–Tanaka and self-consistent methods. Although a large number of studies have been generated to analyze the effective mechanical properties of composites using the homogeneous mass method, few articles have been published on the numerical study of the elastic modulus of concrete using the homogeneous mass method. In this regard, this study mainly focuses on utilizing different homogeneous methods on the basis of fine-scale mechanical modeling and combining the iterative method with multiple homogeneous methods to capture the mechanical relationship between macroscopic and fine-scale. At the same time, it is combined with the software Mathematica (version 14.0.0.0) to simulate the changes and trends of elastic modulus, bulk modulus, shear modulus, and Poisson’s ratio of inclusions and matrix in concrete under different volume ratios and external strain conditions, so as to effectively predict the specific effects of changes in microscale factors on the macroscopic properties of concrete.

## 2. Simulation Methods

In recent years, with the rapid development of computer technology, high-performance calculations have become easier, and the use of finite element meshes at the fine scale to simulate the tiny structural components of macroscopic materials can meet the demand for a large number of calculations of fine mechanics, which directly connects fine mechanics and macroscopic materials. Therefore, in this study, we apply the Dilute-distribution solution method, self-consistent method, generalized self-consistent method, Mori–Tanaka method, differential method, and Voigt and Reuss bounds method with superimposed iterative method to predict the effective elastic modulus of concrete and establish a numerical model of the effective elastic modulus of concrete, which can predict the changes and trends of individual macroscopic properties of the inclusion matrix mixture under the conditions of different volume ratios and externally applied stress or strain at a fine-scale level. In this way, we can predict the changes and trends of the macroscopic properties of the inclusions and matrix at the fine-scale level under different volume ratios and external stresses or strains.

### 2.1. Iterative Method

**Figure 1:**
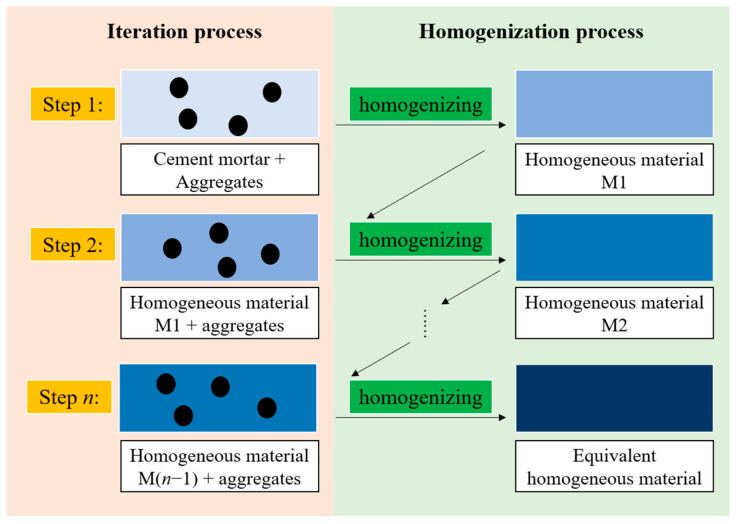
Iterative method model.

The iterative method is used to decompose the inclusions into a number of multiple copies and add them to the matrix unit one by one, and the homogeneous body formed after adding the matrix unit is used as the new matrix for the next iteration until all the inclusions are added. In contrast to the iterative method, the direct method, in which all inclusions are added to the matrix at once to form a homogeneous body, is used [35]. The iterative method, as an efficient means, especially in the context of the development of modern computer technology that provides good conditions for its use, has been widely used in the direction of engineering mathematical problems related to the computational. This method is often used in the direction of the study of the fine mechanics of concrete, and its performance is excellent when combined with Mathematica software for programming and numerical simulation, which can greatly improve the accuracy of numerical simulation. The application of the iterative method to concrete research is shown in Figure 1.

### 2.2. Dilute-Distribution Solution Method

The dilute-distribution solution method (Ds method), a type of homogeneous mass method, assumes that the average strain of the inclusions is equal to the strain of a single inclusion in an infinitely large elastic body. In this method, both the inclusions and the matrix are treated as continuous, homogeneous, isotropic linear elastic materials. The inclusions are randomly distributed in the matrix, and the macroscopic response of the representative volume element (RVE) exhibits isotropy [36].

When considering a two-phase composite material, it is assumed to be an infinite elastomer with a macroscopically uniform strain E applied at infinity. For this composite material, $f_{r},k_{r},\mu_{r},v_{r},(r=1,2)\;$represent the volume fraction of total mixture space occupied by matrix and inclusions, bulk modulus, shear modulus and Poisson’s ratio, respectively, $f_{1}+f_{2}=1$, where inclusions are 1 and matrix is 2.

The effective shear modulus $\mu_{d}$ and effective bulk modulus $k_{d}$ of the composite can be obtained based on the sparse solution method as:

(1) $$k_{d}=k_{2}+\frac{f_{1}\left(k_{1}-k_{2}\right)}{1+\frac{\left(\alpha_{1}-f_{1}\right)}{k_{2}}\left(k_{1}-k_{2}\right)}$$

(2) $$\mu_{d}=\mu_{2}+\frac{f_{1}\left(\mu_{1}-u_{2}\right)}{1+\frac{\left(\beta_{1}-f_{1}\right)}{\mu_{2}}\left(\mu_{1}-\mu_{2}\right)}$$

Among them, the calculation formulas for $\alpha_{1}$ and $\beta_{1}$ are as follows:

(3) $$\;\;\alpha_{1}=\frac{3k_{2}}{3k_{2}+4\mu_{2}}$$

(4) $$\beta_{1}=\frac{6\left(k_{2}-2\mu_{2}\right)}{5\left(3k_{2}-4\mu_{2}\right)}$$

According to Equations (5) and (6), the elastic modulus $E_{d}$ and Poisson’s ratio $v_{d}$ of the material can be obtained.

(5) $$E_{d}=9k_{d}\frac{\mu_{d}}{\mu_{d}+3k_{d}}$$

(6) $$v_{d}=\frac{3k_{d}-2\mu_{d}}{6k_{d}+2\mu_{d}}$$

All modulus values in this formulation are expressed in MPa.

(1) Sparse iteration method

The dilute-distribution solution method is favored for its conceptual simplicity, but the main shortcoming of this method is that it is difficult to ensure the accuracy of calculations in many cases. The main reason for this inaccuracy is that the Dilute-distribution solution method is mainly applicable to the case of a low integral number of entrapped objects, which faces a number of limitations in practical applications. To overcome these limitations, the dilute-distribution solution method is used in combination with the iterative method, which can effectively ensure the accuracy and convergence of the calculation, and this method is called sparse iterative method [37]. In the previous sections, it has been explained in detail how the link between the fine-scale parameters of concrete and its macroscopic mechanical properties can be established through the sparse method modeling. On this basis, the concept of iterative algorithm is introduced and the corresponding computational program is developed in Mathematica software. The model is analyzed by adjusting the relevant conditions and variables for the application of the sparse iterative method.

### 2.3. Mori–Tanaka Method

The Mori–Tanaka method (M-T method) is also known as the effective field method [38,39]. The method was proposed by Mori and Tanaka in the work-hardening of diffusion-hardened materials, and this method fully considers the interaction between the material media. In this method, inclusions are considered to be embedded in an infinitely large matrix, and the far-field stress to which the matrix is subjected is considered to be the average stress of the matrix, rather than directly as an externally applied stress. The reason for this treatment is that each inclusions is practically surrounded by the matrix, and therefore, using the average stress of the matrix to characterize the interaction between the inclusions and the surrounding matrix provides a more reasonable representation of the interactions between the inclusions [40].

Using Mori–Tanaka’s average stress concept of equivalent intercalation principle, the effective bulk modulus and shear modulus of the two-phase composite can be obtained as:

(7) $$\bar{k}=k_{1}\left(1+\frac{f_{1}\left(\frac{k_{2}}{k_{1}}-1\right)}{1+s_{1}\left(1-f_{1}\right)\left(\frac{k_{2}}{k_{1}}-1\right)}\right)$$

(8) $$\bar{\mu }=\mu_{1}\left(1+\frac{f_{1}\left(\frac{\mu_{2}}{\mu_{1}}-1\right)}{1+s_{2}\left(1-f_{1}\right)\left(\frac{\mu_{2}}{\mu_{i}}-1\right)}\right)$$

Among them, the effective bulk modulus and effective shear modulus of composite materials are represented by $\bar{k}$ and $\bar{\mu }$; while $k_{1}$,$\;\mu_{1}$ represent the matrix bulk modulus and shear modulus, respectively; and $k_{2},\;\;\mu_{2}$ represent the intercalation bulk modulus and shear modulus, respectively; $s_{1}=\frac{1+\nu_{1}}{3\left(1-v_{1}\right)},\;s_{2}=\frac{2\left(4-5v_{1}\right)}{15\left(1-v_{1}\right)}$, where $v_{1}$ is the Poisson’s ratio of the matrix and $f_{1}$ is the proportion of the inclusion phase in the total volume of the composite. All modulus values in this formulation are expressed in MPa.

### 2.4. Self-Consistency Method

The self-consistent method (SC method) was originally used to study the elastic properties of polycrystalline materials. Later, two scholars, Hill and Budiansky, extended their application to predict the effective elastic modulus of composite materials [41]. In the self-consistent method, in order to calculate the stress and strain fields inside the inclusions while considering the effects of other inclusions, it is necessary to assume that the inclusions are embedded in an equivalent medium. The elastic constants of this equivalent medium are set to be the overall effective elastic constants of the composite. Therefore, when all the inclusion phases possess the same elastic properties, their effective bulk and shear moduli are, respectively:

(9) $$\bar{k}=k_{1}+\frac{f_{1}\left(k_{2}-k_{1}\right)}{1+S_{1}\left(\frac{k_{2}}{\bar{k}}-1\right)}$$

(10) $$\bar{\mu }=\mu_{1}+\frac{f_{1}\left(\mu_{2}-\mu_{1}\right)}{1+S_{2}\left(\frac{\mu_{2}}{\bar{\mu }}-1\right)}$$

where $\bar{k}$ and $\bar{\mu }$ refer to the effective bulk modulus as well as the effective shear modulus of the composite material, respectively; $k_{1}$, $\mu_{1}$ represent the matrix bulk modulus and shear modulus, respectively; $k_{2},\;\;\mu_{2}$ represent the bulk modulus and shear modulus of inclusion, respectively; $s_{1}=\frac{1+\nu_{1}}{3\left(1-v_{1}\right)},\;s_{2}=\frac{2\left(4-5v_{1}\right)}{15\left(1-v_{1}\right)}$, where $v_{1}$ is the Poisson′s ratio of the matrix and $f_{1}\;$is the proportion of the entrapped phase in the total volume of the composite. All modulus values in this formulation are expressed in MPa.

### 2.5. Generalized Self-Consistent Method

The generalized self-consistent method (GSC method) can be considered as an improved self-consistent method. As an extension and improvement of the traditional self-consistent method, the core idea of the generalized self-consistent method is to consider the inclusions and their surrounding matrix as a whole, and at the same time assume that the whole is embedded in an infinite effective medium. This approach can be analogized to the integration of a simplified representative volume element (RVE) into the overall structure of a composite material. Through the above steps, it can be learned that the generalized self-consistent method takes into account the complexity of the internal structure of the composite material in a more comprehensive way, and thus provides more accurate predictions theoretically. Therefore, the generalized self-consistency method is more reasonable than the self-consistency method, but at the same time, it also increases the difficulty of solving the problem.

For spherical inclusions embedded in an isotropic volume-heavy two-phase composite, the effective bulk modulus of the composite was solved for by Christensen et al. [42]:

(11) $$\bar{k}=k_{1}+\frac{f_{1}\left(k_{2}-k_{1}\right)k_{1}}{k_{1}+\left(1-f_{1}\right)\frac{1+v_{0}}{3\left(1-v_{0}\right)}\left(k_{2}-k_{1}\right)}$$

The analytical equation for the effective shear modulus is more complicated, and its Taylor expansion is as follows:

(12) $$\bar{\mu }=\mu_{1}\left(1+\alpha_{1}f_{1}+\frac{2\left(4-5v_{1}\right)}{15\left(1-v_{1}\right)}\alpha_{1}^{2}f_{1}^{2}+\frac{4\left(4-5v_{1}\right)+54}{225{\left(1-v_{1}\right)}^{2}}\alpha_{1}^{3}f_{1}^{3}-\frac{12}{25{\left(1-v_{1}\right)}^{2}}\alpha_{1}^{3}f_{1}^{\frac{11}{3}}+o\left(f_{1}^{4}\right)\right)$$

Among them:

$$\alpha_{1}=\frac{\mu_{2}-\mu_{1}}{\mu_{1}+\frac{2\left(4-5v_{1}\right)}{15\left(1-v_{1}\right)}\left(\mu_{2}-\mu_{1}\right)}$$

The effective bulk modulus and effective shear modulus of the composite are represented by $\bar{k}$ and $\bar{\mu }$, respectively; $k_{1}$ and $\mu_{1}$ represent the matrix bulk modulus and shear modulus, respectively; $k_{2}$ and $\mu_{2}$, respectively, represent the bulk modulus and shear modulus of the inclusion; $v_{1}$ is the Poisson′s ratio of the matrix, and $f_{1}$ is the proportion of inclusions in the total volume of the composite material. All modulus values in this formulation are expressed in MPa.

### 2.6. Differential Method

In the study of the mechanical properties of composite materials, McLaughlin played a pivotal role in advancing the application of the differential method. This method involves removing a small volume element δ from a homogeneous base material with an initial volume $V_{0}$ and initial stiffness tensor $L_{0}$, and then replacing it with an inclusion of equal volume. By this approach, the effective stiffness increment of the material after homogenization, expressed as $L_{0}+\delta L$, can be calculated. By repeatedly performing this process until the volume fraction of the inclusions reaches a specific ratio, a mathematical model can be gradually developed to describe the relationship between the effective elastic modulus of the composite material, the volume fraction of inclusions, and the elastic properties of both the inclusions and the matrix. The differential equations for the effective bulk modulus and effective shear modulus of spherical two-phase composites are respectively:

(13) $$\frac{d\bar{k}}{df}=\frac{\left(k_{2}-\bar{k}\right)\left(3\bar{k}+4\bar{\mu }\right)}{\left(1-f\right)\left(3k_{2}+4\bar{\mu }\right)}$$

(14) $$\frac{d\bar{\mu }}{df}=\frac{5\bar{\mu }\left(\mu_{2}-\bar{\mu }\right)\left(3\bar{k}+4\bar{\mu }\right)}{\left(1-f\right)\left[\left(9\bar{k}+8\bar{\mu }\right)+6\mu_{2}\left(\bar{k}+2\bar{\mu }\right)\right]}$$

In this context, $\bar{k}$ and $\bar{\mu }$ represent the effective bulk modulus and effective shear modulus of the composite material, respectively; $k_{2}$ and $\mu_{2}$ denote the bulk modulus and shear modulus of the inclusion phase, respectively; f is the volume fraction of the inclusion phase in the total volume of the composite material. The initial conditions are: ${\left.\bar{k}\right|}_{f=0}=k_{1},{\left.\bar{\mu }\right|}_{f=0}=\mu_{1}$, where $k_{1}$ and $\mu_{1}$ represent the bulk modulus and shear modulus of the matrix phase, respectively. All modulus values in this formulation are expressed in MPa.

### 2.7. Voigt Method and Reuss Method

**Figure 2:**
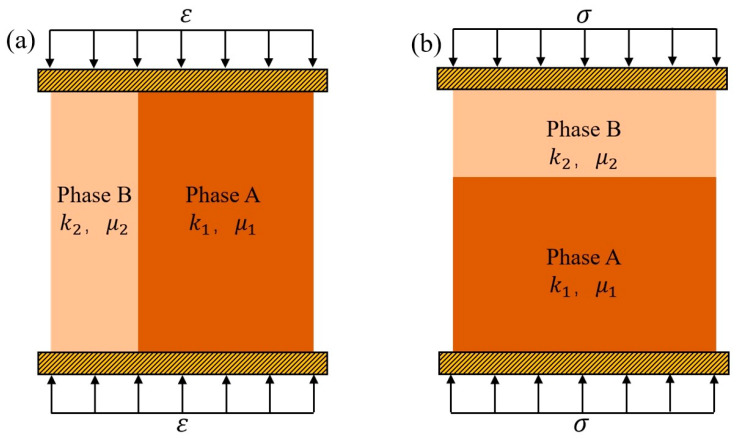
(**a**) Voigt strain model, and (**b**) Reuss stress model.

Voigt proposed the equal-strain model to predict the elastic modulus of composite materials, with the prerequisite assumption that both phases undergo the same strain during deformation [43]. As shown in Figure 2, under this model, the equivalent elastic modulus of the composite material is given by the following formula:

(15) $$\bar{k}=f_{1}k_{1}+\left(1-f_{1}\right)k_{2}$$

(16) $$\bar{\mu }=f_{1}\mu_{1}+\left(1-f_{2}\right)\mu_{2}$$

Here, $\bar{k}$ and $\bar{\mu }$ represent the effective bulk modulus and effective shear modulus of the composite material, respectively; $k_{1}$ and $\mu_{1}$ represent the bulk modulus and shear modulus of the matrix phase, respectively; $k_{2}$ and $\mu_{2}$ represent the bulk modulus and shear modulus of the inclusion phase, respectively; and $f_{1}$ denotes the volume fraction of the inclusion phase. All modulus values in this formulation are expressed in MPa.

To predict the elastic modulus of composite materials, Reuss also proposed the equal-stress model, with the prerequisite assumption that both phases experience the same stress during deformation, as shown in Figure 2 [44]. Under this model, the equivalent elastic modulus of the composite material is given by the following formula:

(17) $$\frac{1}{\bar{k}}=\frac{f_{1}}{k_{1}}+\frac{1-f_{1}}{k_{2}}$$

(18) $$\frac{1}{\bar{\mu }}=\frac{f_{1}}{\mu_{1}}+\frac{1-f_{1}}{\mu_{2}}$$

Here, $\bar{k}$ and $\bar{\mu }$ represent the effective bulk modulus and effective shear modulus of the composite material, respectively; $k_{1}$ and $\mu_{1}$ represent the bulk modulus and shear modulus of the matrix phase, respectively; $k_{2}$ and $\mu_{2}$ represent the bulk modulus and shear modulus of the inclusion phase, respectively; and $f_{1}$ is the volume fraction of the inclusion phase in the total volume of the composite material. All modulus values in this formulation are expressed in MPa.

### 2.8. Overview of Mathematica Software and Its Application in This Study

Mathematica, developed by Wolfram Research, is a comprehensive computational software widely recognized for its advanced symbolic computation capabilities and high-precision numerical analysis. In this study, Mathematica was utilized to construct and implement the mathematical model of the homogenization method for simulating the mechanical behavior of composite materials. Its powerful symbolic engine significantly simplifies the derivation of complex equations involved in the calculation of effective properties, while its numerical capabilities support iterative updating and convergence analysis. The integration of symbolic derivation and numerical simulation within a single platform greatly enhances the efficiency and coherence of the entire modeling and analysis workflow.

**Figure 3:**
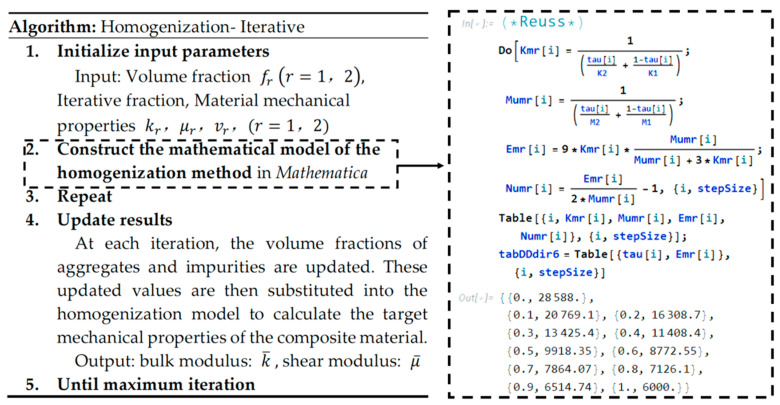
Homogenization-iterative method: algorithmic framework.

This study implements the homogenization method and an iterative enhancement of the homogenization approach using Mathematica to numerically predict the effective mechanical properties of composite materials. The iterative method improves the traditional homogenization approach by continuously updating the volume fractions of constituent phases, enabling the model to more accurately reflect changes in the material’s microstructure and thereby improve simulation accuracy. The detailed implementation process is illustrated in Figure 3.

## 3. Simulation Results

### 3.1. Influence of Aggregate Volume Fraction

#### 3.1.1. Analysis of Stock Test Case Examples

In the study of the simulation methods for the elastic modulus of concrete, case studies are an indispensable part. Through case analysis, the accuracy and feasibility of the analytical methods can be verified. At the same time, case studies can integrate knowledge and methods from different disciplines, such as computer technology, to improve the accuracy of concrete performance predictions. Moreover, by applying theoretical values to specific case studies, the advantages and limitations of different calculation methods, as well as their applicable scope, can be determined, providing a basis for practical applications.

When studying the related parameters of concrete materials, researchers both domestically and internationally often use Stock test data [45] as a reference. Therefore, in this paper, Stock test data are also selected for comparison with the effective elastic modulus of concrete predicted using various homogenization methods, in order to further analyze the application scope and advantages and disadvantages of different homogenization methods.

It should be noted that the aggregate volume ratio refers to the proportion of the total volume occupied by the aggregate phase in the concrete mixture, which includes both the cement matrix phase and the aggregate phase. When the aggregate volume ratio is 1, the mixture consists entirely of the aggregate phase, with no cement matrix phase. Conversely, when the ratio is 0, the mixture consists only of the cement matrix phase, with no aggregates.

In the Stock test, the parameters used are as follows: the aggregate (irregular smooth flint gravel) particle size range varies from 0.15 mm to 19 mm, with the aggregate elastic modulus $E_{g}$ = 74.5 GPa and Poisson’s ratio $v_{g}$ = 0.15; the particle size distribution of the aggregate follows the Fuller distribution law; the elastic modulus of the cement paste $E_{m}$ = 13.4 GPa; and Poisson’s ratio $v_{m}$ = 0.25. It is assumed that the interface layer thickness is zero. Based on the scope of this study and with reference to relevant literature, the parameters of the concrete’s micro-scale binary materials are shown in Table 1. When the aggregate volume fractions are 20%, 40%, 60%, and 80%, the corresponding experimental values of the concrete elastic modulus are 15.8 GPa, 23.2 GPa, 30.7 GPa, and 39.1 GPa, respectively.

#### 3.1.2. Validation of Various Micromechanical Direct Methods

The parameter values of the concrete’s micro-scale two-phase materials listed in Table 1 are substituted into the different homogenization methods mentioned earlier to calculate the predicted simulation values of the concrete’s effective elastic modulus. The calculations are assisted by Mathematica software. To differentiate the influence of the direct method and the iterative method on the predicted simulation values of the effective elastic modulus, the iterative method and the direct method are calculated separately and then compared. It should be noted that in this section, all the micro-mechanics methods are based on the direct method, and the results obtained by combining micro-mechanics and iterative methods will be also discussed.

**Figure 4:**
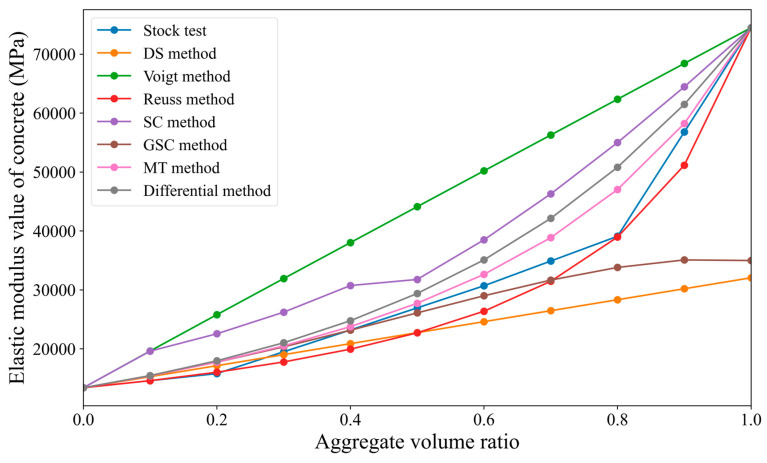
Comparison of the predicted results from various micromechanical direct methods with the Stock test results.

Since the calculation results are relatively complex, this study presents the results in the form of tables and line graphs, as shown in Table 2 and Figure 4. In Figure 4, the aggregate volume fraction is taken as the independent variable on the X-axis, and the overall elastic modulus of the concrete is taken as the dependent variable on the Y-axis. A smooth curve is used to connect the data points.

From Figure 4, it can be observed that (1) overall, the elastic modulus of concrete generally increases with the increase in the aggregate volume fraction, showing an upward trend. (2) When the aggregate volume fraction is below 60%, the results from the sparse method, differential method, generalized self-consistent method, Mori–Tanaka method, and Reuss bound method are in good agreement with the results of the Stock test, with the relative deviation from the Stock test being within 20%; additionally, the lower the aggregate volume fraction, the smaller the relative deviation from the Stock test. (3) When the aggregate volume fraction exceeds 60%, the predicted values of the concrete elastic modulus calculated by most methods deviate significantly from the Stock test results. Only the prediction from the Reuss bound method is close to the Stock test values, with a relative deviation of only 0.4% when the aggregate volume fraction is 80%. (4) The Voigt bound method and self-consistent method show significant differences from the Stock test at all aggregate volume fractions, consistently being slightly higher than the Stock test values.

#### 3.1.3. Validation of Various Micromechanical Iterative Methods

**Figure 5:**
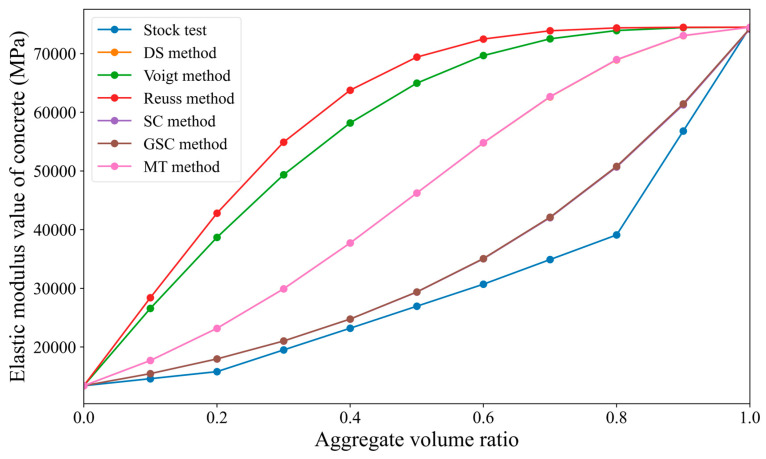
Comparison of the predicted results from various micromechanical iterative methods with the Stock test results.

Similar to Section 3.1.2, the parameter values of the concrete’s micro-scale two-phase materials listed in Table 1 are substituted into the different homogenization methods mentioned earlier. However, when incorporating the parameters, the principle of the iterative method is applied, i.e., the inclusion units are decomposed into several smaller parts, and each part is added to the matrix unit. The homogenized body formed after adding a matrix unit is then used as the new matrix for the next iteration, until all inclusions are added. The final results, compared with the known Stock test results, are shown in Table 3. In order to visually reflect the trend of the concrete’s elastic modulus with respect to the aggregate volume fraction, the values in the table are plotted as a line graph, as shown in Figure 5.

From Figure 5, it can be observed that (1) overall, the elastic modulus of concrete generally increases with the increase in the aggregate volume fraction, showing an upward trend. (2) Unlike the direct method, the elastic modulus prediction values obtained by combining various micro-mechanical methods with the iterative method are all greater than the Stock test values. (3) The predicted values obtained by the sparse method and the Mori–Tanaka method when combined with the iterative method are very close. Additionally, when the aggregate volume fraction f < 60%, the predicted values of the self-consistent method and generalized self-consistent method are quite close to the Stock test values. Even when the aggregate volume fraction is 60%, the relative deviation from the Stock test is only 14%. (4) It is evident that the Voigt method and the Reuss method, when combined with the iterative approach in numerical simulations, exhibit large deviations from the case study results. Therefore, they are no longer suitable for predicting the elastic modulus of concrete in conjunction with the iterative method. In contrast, the iterative method yields substantial improvements for the GSC method and SC method, reducing the maximum prediction error from 53.04% to 29.89% compared to the non-iterative results, thus significantly enhancing their predictive capability.

### 3.2. Analysis of the Influence of Different Types of Aggregates on Cement Matrix

#### 3.2.1. The Influence of Different Types of Aggregates

In Section 3, this paper uses the Stock test to verify the prediction accuracy of each method under different aggregate volume fractions. In this chapter, the focus will be on exploring the prediction accuracy of each method when the aggregate types are different. In reference [46], the authors F.P. Zhou et al. studied the influence of different types of coarse aggregates on the elastic modulus of high-performance concrete. The experiments in this paper used six different materials as aggregates, with the elastic modulus of the aggregates ranging from 5.2 GPa to 210 GPa. This broad range meets the requirements for the case study in this chapter. Additionally, the reference provides a constant aggregate volume fraction of 0.425, and the elastic modulus of the cement matrix is 40.8 GPa. The Poisson’s ratio of both the aggregate and the cement matrix is taken as the measured value for concrete samples cured for 28 days. The values of the elastic modulus and Poisson’s ratio for each material are provided in the referenced work; the specific reference values are shown in Table 4.

**Figure 6:**
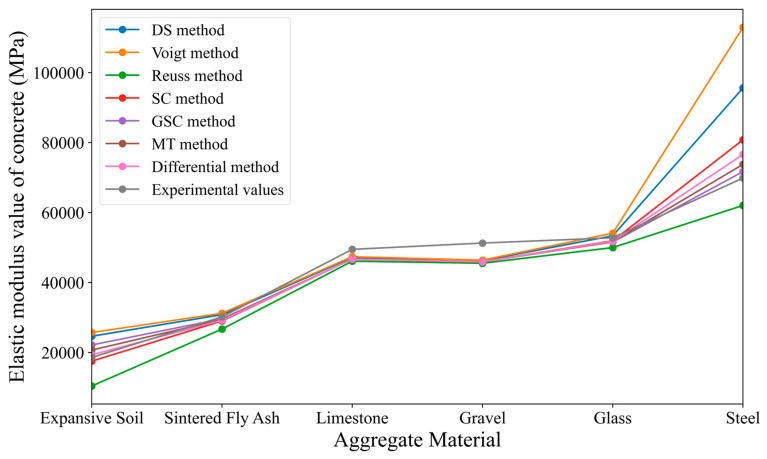
Comparison between predicted results from micromechanical approaches and case study results.

Firstly, by combining the reference values of aggregate and cement matrix provided in the example, the elastic modulus of concrete is predicted using the direct methods of micro-mechanics described in introduction. The predicted values are then compared with the measured elastic modulus values of concrete given in the example. The main experimental data and predicted data are shown in Table 5. Based on the data in Table 5, Figure 6 is plotted, and the relative error is calculated, with the results presented in Table 6. In Figure 6, the horizontal axis represents six types of aggregate materials: expansive clay, sintered fly ash, limestone, gravel, glass, and steel, numbered from 1 to 6.

From Table 5 and Figure 6, it can be concluded that: (1) When the aggregate is harder than the cement matrix, the elastic modulus of concrete is higher than that of the cement matrix but lower than that of the aggregate. Conversely, when the aggregate is softer than the cement matrix, the elastic modulus of concrete is lower than that of the cement matrix but higher than that of the aggregate. (2) When the aggregate is soft, such as expansive clay as an inclusion, the predicted values from various micromechanical methods show significant deviations from the experimental values. Similarly, when the aggregate is hard, such as steel as an inclusion, most micromechanical methods also produce results with considerable deviation. However, when the elastic modulus of the aggregate is close to that of the cement matrix, the predictions from micromechanical methods are relatively accurate, with the relative error typically controlled within ±10%. (3) Among all the methods, the predictions using the differential method are the closest to the experimental values of the example. However, the calculation procedure of the differential method is quite complicated, making it less suitable for large-scale applications. Nevertheless, the differential approach provides relatively better accuracy and is particularly useful in specific cases. (4) Conceptually, the generalized self-consistent method is an improvement on the self-consistent method. When the ratio of the elastic modulus of the aggregate to that of the cement matrix is neither very large nor very small, the comparison results show that the generalized self-consistent method provides more accurate predictions than the self-consistent method. However, when there is a significant disparity between the elastic moduli of the aggregate and the cement matrix, the predictions from both methods are somewhat unsatisfactory.

**Figure 7:**
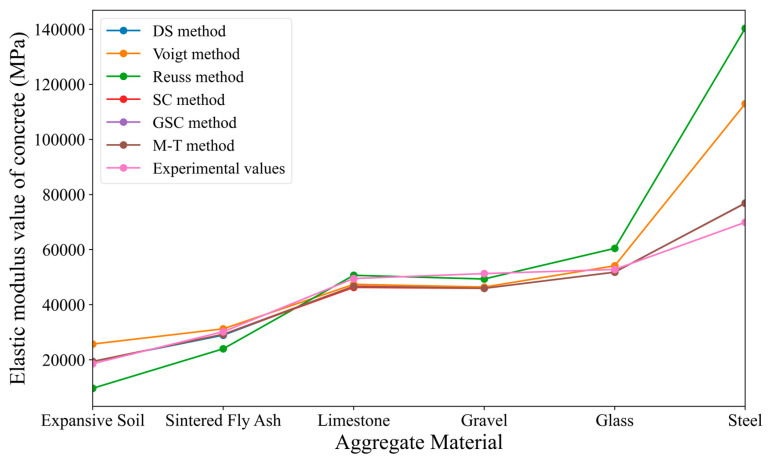
Comparison of predicted results from various micromechanical iterative methods with case study results.

As in the previous section of this study, the aggregate and cement matrix test values listed in Table 4 are substituted into the various homogeneous methods mentioned earlier. At the same time, the iterative method principle is applied when inputting the parameters, that is, the inclusion unit is decomposed into several parts, which are then added to the matrix unit one by one. The homogeneous body formed after adding the matrix unit is used as the new matrix for the next iteration, until all inclusions are incorporated. The final predicted results using the micromechanical method are shown in Table 7, along with the known case study results. To more intuitively reflect the variation trend of the concrete elastic modulus with respect to the aggregate volume ratio, the values in the table are plotted as a line graph, as shown in Figure 7. It should be noted that the horizontal axis in Figure 7, labeled 1 to 6, represents six types of aggregate materials: expansive clay, sintered fly ash, limestone, gravel, glass, and steel, arranged in ascending order of their elastic modulus values.

Based on Table 7 and Table 8, and Figure 7, and in conjunction with Table 5 and Table 6, and Figure 6, the following conclusions can be drawn: (1) For micro-mechanical methods, the accuracy after incorporating the iterative method is significantly improved compared to directly applying the micro-mechanical methods, especially when the elastic modulus of the aggregate is close to that of the cement matrix. (2) When the difference between the elastic modulus of the aggregate and the bulk elastic modulus of the cement is too large, both the Voigt and Reuss bounds fail to predict the elastic modulus of concrete effectively. (3) When the iterative method is used, the results of the M-T method, DS method, SC method, and the GSC method are all very close, and they all predict the elastic modulus of concrete quite accurately. The incorporation of the iterative method improves the prediction accuracy of the GSC, SC, and M-T methods, reducing the maximum error from 15.6% to 10.43% and thereby enhancing overall model performance.

#### 3.2.2. Impact of Different Types of Cement Matrix

When discussing the potential impact of different types of cement matrix on the predicted results, this study uses the experimental data from Chapter 4 of reference [47] as experimental values for comparison with the predicted values from various micromechanical methods. This is performed to examine the influence of different types of cement matrix on the predicted values. The experimental results provide the elastic moduli of three different matrix phases, which are 14.66 GPa, 20.34 GPa, and 23.56 GPa, respectively. In addition, the volume fraction of coarse aggregates in the tests was consistently 45%, and the elastic modulus of the coarse aggregates was 74.5 GPa. The specific parameters are listed in Table 9.

**Figure 8:**
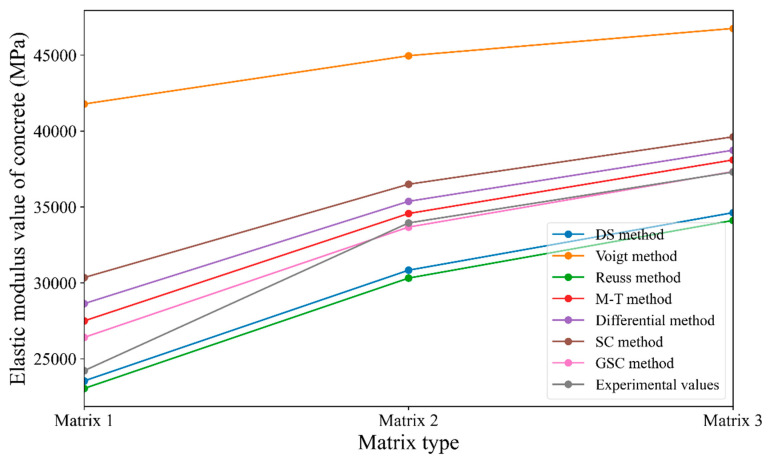
Comparison of predicted results from various micromechanical direct methods with case study results.

Similar to Section 3.2.1, the reference values from Table 9 are substituted into the various micromechanical methods mentioned earlier to obtain the predicted values, which are then compared with the known experimental values from the case study. This comparison helps assess the prediction accuracy of each micromechanical method. The predicted and experimental values are shown in Table 10. Additionally, based on the data in Table 10, a line graph (Figure 8) is created to more intuitively reflect the prediction accuracy of the different micromechanical methods. In Figure 8, the symbols Matrix 1, Matrix 2, and Matrix 3 on the horizontal axis represent three different cement matrix phases, with corresponding elastic moduli of 14.66 GPa, 20.34 GPa, and 23.56 GPa, respectively.

Based on Table 10 and Figure 8, the following conclusions can be drawn: (1) With the exception of the Voigt method, the errors between the predicted values and the experimental values for the other micromechanical methods are relatively small. (2) The Voigt and Reuss methods exhibit significant errors in predicting the concrete elastic modulus when there is a large discrepancy between the matrix elastic modulus and the aggregate elastic modulus. This case study also validates the conclusion of Section 3.2.1. (3) Among all the methods, the predicted values from the Mori–Tanaka method are closest to the experimental values, possibly because the Mori–Tanaka method considers the interaction of the media by utilizing the changes in stress and strain at a distance. The accuracy is higher when the aggregate is relatively hard and the aggregate volume ratio is not large.

**Figure 9:**
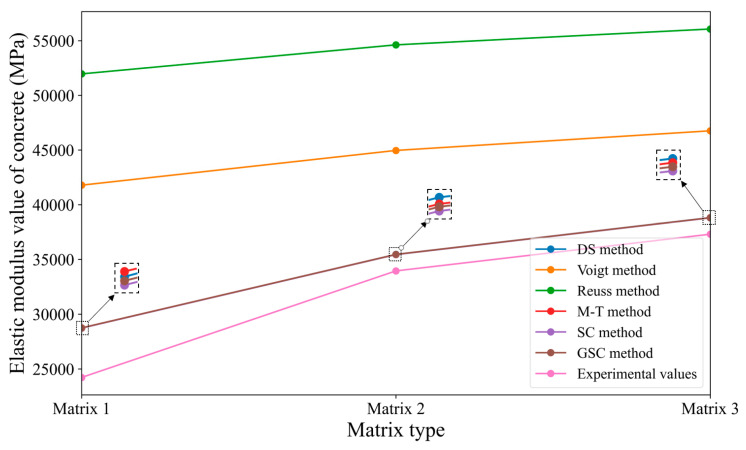
Comparison of predicted results from various micromechanical iterative methods with case study results.

Similar to Section 3.2.1, the specific values provided in Table 9 are applied to the various micromechanical methods discussed earlier. When introducing the parameters, the iterative method strategy is used: the inclusion phase is divided into smaller parts, and these smaller parts are gradually added to the matrix phase. After each iteration, the newly formed homogeneous body is treated as the matrix for the next iteration. This process is repeated until all inclusion phases are fully integrated into the matrix. The final predicted results from the micromechanical methods and the known case study results are shown in Table 11. To more intuitively reflect the prediction accuracy of the different micromechanical methods under varying matrix conditions, the values in the table are plotted as a line graph, as shown in Figure 9. It is worth noting that the predicted values obtained from the M-T method, SC method, and GSC self-consistent method are quite similar, so the three lines in the graph almost overlap.

From Table 11 and Figure 9, combined with Table 10 and Figure 8, the following conclusions can be drawn:(1) The predicted concrete elastic modulus values from the Voigt and Reuss methods show significant discrepancies with the experimental values. This is due to a large difference between the matrix elastic modulus and the aggregate elastic modulus. (2) Compared to the direct methods, the prediction accuracy of the M-T method, the SC method, and the GSC method have been improved to varying degrees after applying the iterative approach. In particular, the GSC method and SC method exhibit notable enhancements: the prediction error range is reduced from an initial 14.42–25.33% (without iteration) to a narrower range of 4.01–18.59% after iteration. This indicates that the iterative method significantly improves the predictive performance of these homogenization approaches. (3) As the difference between the matrix elastic modulus and the aggregate elastic modulus increases, the prediction errors of the homogeneous methods also increase. As the matrix elastic modulus approaches the aggregate elastic modulus, the predicted values from the homogeneous methods become closer to the experimental values. (4) Based on the results of this case and the two previous examples, it can be observed that the predictive performance of the Voigt and Reuss methods is generally unsatisfactory. This is primarily due to the simplified assumptions inherent in these models: the Voigt method assumes that all phases experience the same stress in the loading direction, while the Reuss method assumes uniform strain across all phases in the transverse direction. Although these assumptions may be valid for isotropic and highly homogeneous materials, they become problematic when applied to real-world composites with complex microstructures. In particular, when there are significant stiffness contrasts between constituent phases, these assumptions can lead to considerable overestimation or underestimation of the effective elastic modulus, thereby compromising the overall predictive accuracy of the models.

## 4. Conclusions and Discussion

### 4.1. Conclusions

The elastic modulus of concrete is a key parameter in structural design, influencing deformation control and crack resistance. Due to concrete’s heterogeneous multiphase nature, accurately predicting its elastic modulus is challenging. This study systematically compares several classical homogenization methods and introduces an innovative iterative optimization strategy that dynamically updates model parameters to better capture complex multiphase interactions. The proposed approach is validated with two independent experimental datasets, showing notable improvements in prediction accuracy and applicability. Methods used include the DS method, SC method, GSC method, M-T method, differential method, Voigt method, and Reuss method, all enhanced by the iterative strategy.

To validate the effectiveness of the proposed approach, two independent sets of experimental data from the literature are used for comparative analysis. The first experimental set features aggregate (irregular smooth flint gravel) with a relatively high elastic modulus (74.5 GPa) and a lower matrix modulus (13.4 GPa), with a wide variation in aggregate volume fraction. The second set spans a broader range, with aggregate (expansive clay, sintered fly ash, limestone, gravel, glass, steel) elastic moduli varying from 5.2 GPa to 210 GPa, including cases where the aggregate modulus is either higher or lower than that of the cement matrix. The results indicate notable differences among the methods in terms of theoretical assumptions and applicable conditions, leading to varying levels of prediction accuracy. For instance, the DS method assumes that the strain in the inclusions is equal to the strain they would experience in an infinite matrix. It is suitable for cases with low aggregate volume fractions and is computationally simple, but its accuracy significantly decreases as the volume fraction increases; the M-T method considers interphase stress transfer mechanisms and performs well with stiff aggregates at low volume fractions; the SC and GSC methods offer a more comprehensive description of interphase interactions, making them appropriate when the moduli of the matrix and aggregate are similar or when aggregate stiffness is high; the Voigt and Reuss methods are suitable for quick estimations but tend to yield larger errors; and although the Differential method is computationally complex, it demonstrates strong predictive performance under specific conditions.

The iterative improvement strategy proposed in this study refines the simulation process by allowing model parameters to update dynamically, better capturing the complex interactions within multiphase materials and significantly improving predictive accuracy. Comparative analysis shows that the prediction performance of the GSC, SC, and M-T methods improves substantially with the introduction of iteration. In three representative cases, the maximum prediction errors decreased from 53%, 15.62%, and 25% to 30%, 10.43%, and 16%, respectively, confirming the effectiveness of the proposed strategy.

Furthermore, this study reveals that the aggregate volume fraction is a key factor influencing prediction accuracy. When the volume fraction increases from 20% to 60%, the prediction error can increase by up to 20%. Under conditions of high aggregate stiffness (>50 GPa) and low volume fraction (<40%), the GSC and M-T methods, when combined with the iterative strategy, show particularly strong performance. The DS method also performs well at low-volume fractions, while the Differential Method dynamically accounts for local interactions, thus improving the overall predictive accuracy of mechanical behavior.

In summary, different micromechanical methods have distinct advantages, and their predictions are generally more reliable when the aggregate volume fraction is low and the moduli of the matrix and aggregate are close. The “iterative + homogenization” strategy proposed in this study significantly improves the prediction accuracy of various models in complex material systems such as concrete, with particularly notable enhancements observed for the GSC, SC, and M-T methods. This provides a new perspective and theoretical support for performance modeling of composite materials.

### 4.2. Discussion

In this study, various classical homogenization methods were employed to numerically simulate the elastic modulus of concrete, and an iterative strategy was introduced to enhance prediction accuracy. The simulation results indicate that this “iteration + homogenization” approach significantly improves the model’s predictive capability for the mechanical properties of concrete, particularly under conditions involving complex material compositions or substantial stiffness contrast between phases. However, the methodology still relies on several simplifying assumptions that may introduce deviations in the context of concrete’s intricate microstructure, thereby affecting the accuracy and applicability of the model.

First, this study assumes concrete to be a typical two-phase composite system composed of aggregates and cementitious matrix, while neglecting the presence of the interfacial transition zone (ITZ). This modeling simplification facilitates computational analysis but excludes a structural region that critically influences the overall stiffness in real concrete. The ITZ typically exhibits lower elastic modulus and higher porosity, along with spatial heterogeneity in thickness and mechanical properties. Under high aggregate volume fractions, this assumption may result in systematic underestimation or overestimation of local stress transfer capabilities. Incorporating the ITZ as an independent phase in future models is expected to improve the physical fidelity of predictions.

Second, the model presumes both aggregates and matrix to be isotropic and homogeneous, whereas the actual microstructure of concrete is considerably more complex. Aggregates often exhibit irregular shapes and a wide particle size distribution, while the matrix may contain pores, microcracks, and other non-uniform features. These microstructural characteristics can induce stress concentrations and significantly influence the material’s deformation response and failure behavior. Neglecting such heterogeneities inevitably constrains the model’s ability to represent the true mechanical behavior of concrete. Introducing parameters such as aggregate shape factors, orientation distributions, or porosity may enhance the model’s accuracy in capturing stress transfer and localized responses.

Moreover, in the experimental validation phase, certain benchmark datasets assumed a fixed aggregate volume fraction, which to some extent limits the model’s generalizability across varying phase compositions. In practice, variations in aggregate volume fraction have a pronounced impact on the macroscopic stiffness of concrete, especially when there is a large stiffness mismatch between aggregates and matrix. This nonlinearity becomes particularly evident under such conditions. Therefore, conducting systematic modeling and experimental verification across a broader range of aggregate contents will help comprehensively assess the model’s applicability and sensitivity.

In summary, the proposed iterative optimization-based homogenization strategy achieves a notable enhancement in prediction accuracy while preserving the computational efficiency of classical models, demonstrating its practical potential in modeling complex composite materials. Nonetheless, to further improve the model’s adaptability in engineering contexts, future research may expand in three directions: (1) incorporating explicit modeling of the ITZ to better capture localized weak zone behavior; (2) integrating heterogeneity parameters of aggregates and matrix to more realistically reflect microstructural effects on macroscopic performance; and (3) conducting dynamic modeling and validation across diverse volume fractions and material contrasts to establish a more robust predictive framework. These advancements will provide a more reliable theoretical basis for the performance-oriented design of high-performance concrete with multiscale mechanical modeling.

## Figures and Tables

**Table 1:** Reference values for two-phase materials in concrete microstructure.

Two-Phase Materials	Elastic Modulus /GPa	Poisson’s Ratio
Aggregate phase	74.5	0.15
Cement matrix phase	13.4	0.25

**Table 2:** Prediction values of concrete elastic modulus by direct micromechanics method and Stock test values.

Aggregate Volume Ratio	$\mathrm{Elastic}\;\mathrm{Modulus}\;\mathrm{Value}\;\mathrm{of}\;\mathrm{Concrete}\;E_{d}$/MPa							
	Stock Test	DS Method	Voigt Method	Reuss Method	SC Method	GSC Method	M-T Method	Differential Method
0	13,400	13,400	13,400	13,400	13,400	13,400	13,400	13,400
0.1	/	15,274	19,635	14,597	19,608	15,417	15,414	15,464
0.2	15,800	17,144	25,798	16,029	22,558	17,736	17,751	17,969
0.3	/	19,012	31,925	17,773	26,221	20,340	20,496	21,025
0.4	23,200	20,879	38,031	19,942	30,742	23,165	23,766	24,774
0.5	/	22,744	44,124	22,714	31,776	26,103	27,730	29,392
0.6	30,700	24,608	50,209	26,382	38,498	29,005	32,635	35,092
0.7	/	26,472	56,287	31,462	46,294	31,667	38,863	42,135
0.8	39,100	28,334	62,361	38,966	55,009	33,817	47,034	50,821
0.9	/	30,196	68,432	51,169	64,465	35,086	58,227	61,488
1	74,500	32,058	74,500	74,500	74,500	34,986	74,500	74,500

**Table 3:** Predicted values of concrete elastic modulus using various mesomechanical iterative methods compared with Stock experimental values.

Aggregate Volume Ratio	$\mathrm{Elastic}\;\mathrm{Modulus}\;\mathrm{Value}\;\mathrm{of}\;\mathrm{Concrete}\;E_{d}$/MPa						
	Stock Test	DS Method	Voigt Method	Reuss Method	SC Method	GSC Method	M-T Method
0	13,400	13,400	13,400	13,400	13,400	13,400	13,400
0.1	/	17,697	26,586	28,414	15,463	15,464	17,695
0.2	15,800	23,186	38,689	42,804	17,965	17,968	23,179
0.3	/	29,914	49,349	54,910	21,015	21,023	29,905
0.4	23,200	37,732	58,188	63,773	24,753	24,769	37,725
0.5	/	46,239	64,967	69,408	29,353	29,383	46,243
0.6	30,700	54,800	69,670	72490	35,028	35,078	54,819
0.7	/	62,637	72,523	73,,896	42,034	42,114	62,667
0.8	39,100	68,950	73,942	74,388	50,671	50,790	68,981
0.9	/	73,057	74,435	74,493	61,274	61,446	73,077
1	74,500	74,500	74,500	74,500	74,172	74,275	74,500

**Table 4:** Experimental values of aggregates and cement matrix.

Aggregate Material	Elastic Modulus Values of Aggregates (Matrix) *E_d_*/Gpa	Poisson’s Ratio	Volume Fraction	Experimental Values of Concrete Elastic Modulus *E_d_*/Gpa
Expansive Clay	5.2	0.29	0.425	18.6
Sintered Fly Ash	18.2	0.26		30.2
Limestone	56	0.27		49.5
Gravel	54	0.21		51.3
Glass	72	0.25		52.8
Steel	210	0.28		69.9
Cement Matrix	40.8	0.21	0.575	/

**Table 5:** Comparison between experimental and predicted values of concrete elastic modulus.

Aggregate Material	$\mathrm{Elastic}\;\mathrm{Modulus}\;\mathrm{Value}\;\mathrm{of}\;\mathrm{Concrete}\;E_{d}$/MPa							
	DS Method	Voigt Method	Reuss Method	SC Method	GSC Method	MT Method	Differential Method	Experimental Values of Concrete Elastic Modulus/MPa
Expansive Clay	24,663	25,717	10,436	17,541	22,173	20,711	19,345	18,600
Sintered Fly Ash	30,828	31,241	26,706	29,030	29,655	29,433	29,234	30,200
Limestone	47,179	47,392	46,120	46,731	46,666	46,682	46,697	49,500
Gravel	46,277	46,410	45,530	45,985	45,920	45,936	45,945	51,300
Glass	53,373	54,124	50,010	51,933	51,591	51,700	51,800	52,800
Steel	95,632	112,981	62,047	80,818	71,725	73,797	76,633	69,900

**Table 6:** Error between predicted and experimental values of concrete elastic modulus.

Aggregate Type	DS Method	Voigt Method	Reuss Method	SC Method	GSC Method	MT Method	Differential Method
Expansive Clay	32.60%	38.26%	43.89%	−5.70%	19.21%	11.35%	4.01%
Sintered Fly Ash	2.08%	3.45%	11.57%	−3.87%	−1.80%	−2.54%	−3.20%
Limestone	−4.69%	−4.26%	−6.83%	−5.59%	−5.73%	−5.69%	−5.66%
Gravel	−9.79%	−9.53%	11.25%	10.36%	−10.49%	−10.46%	−10.4%
Glass	1.09%	2.51%	−5.28%	−1.64%	−2.29%	−2.08%	−1.89%
Steel	36.81%	61.63%	11.24%	15.62%	2.61%	5.58%	9.63%

**Table 7:** Comparison of concrete elastic modulus experimental and predicted values.

Aggregate Material	$\mathrm{Elastic}\;\mathrm{Modulus}\;\mathrm{Value}\;\mathrm{of}\;\mathrm{Concrete}\;E_{d}$/MPa						
	DS Method	Voigt Method	Reuss Method	SC Method	GSC Method	M-T Method	Experimental Values of Concrete Elastic Modulus/MPa
Expansive Clay	19,421	25,717	9661	19,383	19,400	19,402	18,600
Sintered Fly Ash	28,955	31,241	23,995	29,245	29,250	29,250	30,200
Limestone	46,703	47,392	50,677	46,300	46,701	46,710	49,500
Gravel	45,950	46,410	49,335	45,948	45,948	45,949	51,300
Glass	51,822	54,124	60,457	51,812	51,817	51,817	52,800
Steel	76,911	112,981	140,362	76,805	76,858	76,858	69,900

**Table 8:** Relative error of concrete elastic modulus predicted values relative to experimental values.

Aggregate Material	Error of Elastic Modulus Prediction Value Relative to Experimental Value					
	DS Method	Voigt Method	Reuss Method	SC Method	GSC Method	M-T Method
Expansive Clay	4.41%	38.26%	−48.06%	4.21%	4.30%	4.31%
Sintered Fly Ash	−4.12%	3.45%	−20.55%	−3.16%	−3.15%	−3.15%
Limestone	−5.65%	−4.26%	2.38%	−6.46%	−5.65%	−5.64%
Gravel	−10.4%	−9.53%	−3.83%	−10.43%	−10.43%	−10.43%
Glass	−1.85%	2.51%	14.50%	−1.87%	−1.86%	−1.86%
Steel	10.03%	61.63%	100.80%	9.88%	9.95%	9.95%

**Table 9:** Reference values for the micromechanical two-phase material properties of concrete.

Two-Phase Materials	Elastic Modulus/GPa	Poisson’s Ratio
Aggregate phase	74.5	0.15
Cement matrix phase	14.66	0.25
	20.34	
	23.56	

**Table 10:** Comparison of concrete elastic modulus experimental values and predicted values.

Matrix Type	DS Method	Voigt Method	Reuss Method	M-T Method	Differential Method	SC Method	GSC Method	Experimental Values
Matrix 1	23,547	41,784	23,050	27,499	28,636	30,354	26,414	24,220
Matrix 2	30,838	44,961	30,318	34,570	35,374	36,498	33,677	33,950
Matrix 3	34,627	46,758	34,115	38,100	38,740	39,620	37,330	37,300

**Table 11:** Comparison of concrete elastic modulus experimental values and predicted values.

Matrix Type	DS Method	Voigt Method	Reuss Method	M-T Method	SC Method	GSC Method	Experimental Values
Matrix 1	28,743	41,784	51,964	28,745	28,723	28,742	24,220
Matrix 2	35,461	44,961	54,619	35,457	35,439	35,454	33,950
Matrix 3	38,819	46,758	56,068	38,811	38,795	38,808	37,300

## Data Availability

The original contributions presented in this study are included in the article. Further inquiries can be directed to the corresponding author.
